# Using urine nitrite sticks to test for urinary tract infection in children aged < 2 years: a meta-analysis

**DOI:** 10.1007/s00467-019-04226-6

**Published:** 2019-03-20

**Authors:** Malcolm G. Coulthard

**Affiliations:** Great North Children’s Hospital, Queen Victoria Road, Newcastle upon Tyne, NE1 4LP UK

**Keywords:** Child, Urinary tract infection, Urine, Culture, Bacteria, Diagnosis, Nitrite, Stick testing

## Abstract

**Background:**

This study aimed to determine whether nitrite sticks are as sensitive at detecting urinary tract infection (UTI) in children <2 years as they are in older children.

**Methods:**

I reanalysed data on using nitrite sticks to detect UTIs for children aged either < 2 or 2–18 years. For sensitivity, evidence of a UTI was defined as level 1 when a single uropathogen grew ≥ 10^5^ colony forming units/ml (cfu/ml) in two urine samples, level 2 when just one sample was cultured or a threshold of < 10^5^ cfu/ml was used, and level 3 if mixed growths or *Staphylococcus albus* was considered to be positive. For specificity, children were defined as uninfected if they had 1 sterile urine culture. I also reanalysed our previously published data by age.

**Results:**

The sensitivity was lower for children aged < 2 years (11 studies, 1321 subjects) than for older children (9 studies, 295 subjects), whether the level-1 values or all the studies were analysed (Fisher’s exact test, *p* < 0.0001 for both). The level-1 sensitivities were 0.23 in the infants and 0.81 among older children (odds ratio = 0.07, 95% confidence interval 0.03–0.18). The specificity was very high in infants (10 studies, 1783 cases) and older children (7 studies, 5952 cases), at 0.990 and 0.996.

**Conclusions:**

Nitrite sticks only have a 23% sensitivity in children aged < 2 years, so cannot reliably rule out UTIs. A positive nitrite stick test is about 99% likely to indicate a UTI in children of any age.

## Introduction

Young children with urinary tract infections (UTIs) should be treated promptly with antibiotics to reduce sequelae [1, 2], and ideally within 3 days in infants < 2 years of age to reduce their risk of developing permanent kidney scars [3, 4]. Because urine culture cannot provide immediate screening, it is important to find sensitive point-of-care tests that can identify infected urine samples rapidly and can help to decide whether to undertake a urine culture [5] or to commence antibiotic treatment. Nitrite sticks are the most convenient method of doing this.

Most uropathogens metabolise urinary nitrate to produce nitrite, which is not normally present in urine. This can be detected as a colour change in nitrite sticks if sufficient bacteria are incubated in urine long enough to produce recordable and stable concentrations. Studies consistently report high specificity (few false positives) but record the widest range of sensitivities of any UTI screening test [1]. This is usually ascribed to short bacterial incubation times caused by urinary frequency, while other possibly important causes are ignored [1, 5–9]. Children may not eat enough fruits or vegetables to provide sufficient urinary nitrate [10–12], and their nitrite ions may be unstable if their urine has a pH < 6, a sodium concentration < 40 mmol/l [13], or contains vitamin C [11, 13] or urobilinogen [13]. These biochemical conditions are common in the young due to low salt intakes, high water turnover, and vitamin C supplements. I therefore reanalysed our paper [14] and reviewed other reports to compare nitrite stick testing separately for children under 2 and over 2 years of age.

## Methods

I sourced papers where nitrite sticks were evaluated separately for infants aged < 2 years and children aged 2–18 years, from NICE guidelines [1] and a Medline (1946–August 2018) search, combining ‘urinary tract infection *or* bacteriuria’ *and* ‘nitrite’ in children (≤ 18 years), and followed up earlier papers not included in computerised databases. I excluded children already known to have structural urinary tract abnormalities and included studies which compared urine collection methods in healthy individuals as well as those with a clinical suspicion of UTI. I also reanalysed the data our group has previously published on nitrite stick testing [14] to look for sex or age effects.

I recalculated the sensitivity results using the following diagnostic culture criteria for UTIs: quality level 1 = a single uropathogen in two urine samples at ≥ 10^5^ colony forming units/ml (cfu/ml); level 2 = single uropathogen in one sample at ≥ 10^5^ cfu/ml or two samples at < 10^5^ cfu/ml; level 3 = mixed growths or skin commensals counted as positive. For specificity, children were considered infection free if they had at least one sterile urine, that is one which grew no bacteria in a petri dish (< 10^3^ cfu/ml). I tested for differences by age group using unpaired *t* tests (t) for individual-study data, and either *χ*^2^ or Fisher’s exact test (F) for pooled data, according to the dataset size. I compared the sensitivity odds ratios using a forest plot.

I produced leaf plots to demonstrate the usefulness of using nitrite stick tests for infants and older children [15]. These graphs are constructed from the sensitivity and specificity data of diagnostic tests and show the range of pre-test probabilities (how suspicious you were that the child had a UTI) along the diagonal ‘vein’ of the leaf. For any level of pre-test estimate of probability, the impact of a positive test result on that probability is shown by the vertical height upwards to the red line and the impact of a negative test is shown as the fall to the blue line below. A poor test will produce a narrow willow-type leaf pattern, while a powerful test will produce a broad-leaf that nearly reaches the corners of the plot. New plots can be constructed for any diagnostic test data by accessing the free website www.childhealthafrica.org.

## Results

### Material

Eighteen papers provided sufficient age group–specific data to allow me to calculate their sensitivity or specificity values for infants < 2 years or older children separately, of which two enrolled children from both age groups [14, 16], and one included two sub-sets of older children [17], resulting in a total of 21 informative data sets (Table 1). Eleven studies provided sensitivity data for infants [14, 16, 18–26], and eight for older children [12, 14, 16, 17, 27–30], while ten papers provided specificity data for infants [14, 16, 19, 21–26, 31], and seven for older children [14, 16, 17, 27–30].

**Table 1:** Sensitivity and specificity values for nitrite stick testing, calculated for individual studies, presented in date order

				Sensitivities			Specificities		
Reference and year		Reason for study	Age range	Sensitivity^a^	Evidence Q-level^b^	Valid cases (*n*)	Specificity^a^	Evidence Q-level^c^	Valid cases (*n*)
Infants < 2 years									
31	1985	Screening	3–28 months				0.993	1	439
18	1991	?UTI	<1.5 year	0.16	3	37			
19	1993	Screening	Newborns	0.17	1	24	0.986	1	223
20	1998	?UTI	<1 year	0.447	2	237			
16	1998	?UTI	<1 year	0.1	2	7	0.99	1	115
21	2001	?UTI	<4 years	0.24	2	21	1.000	1	230
22	2002	?UTI	<2 years	0.35	2	23	0.98	1	170
23	2008	?UTI	<2 years	0.47	2	32	0.97	1	29
14	2010	?UTI	<2 years	0.5	1	6	0.96	1	45
24	2014	?UTI	Median 6.2 months	0.52	2	42	0.993	1	300
26	2015	?UTI	<3 months	0.395	2	243	1.00	1	115
25	2015	?UTI	<3 months	0.371	2	649	0.99	1	117
Older children									
27	1974	Screening	8–11 years	0.92	1	25	0.998	1	3494
28	1977	?UTI	Pre-school	0.70	1	42	1.00	1	55
12	1978	?UTI	2–18 years	0.93	1	30			
29	1978	Screening	5–12 years	0.60	1	15	1.000	1	1043
17	1982	?UTI	2–15 years	0.56	3	101	0.999	1	923
17	1982	?UTI	2–15 years	0.88	3	41			
30	1998	Screening	5–14 years	0.60	2	15	0.98	1	171
16	1998	?UTI	1–18 years	0.6	2	9	0.96	1	194
14	2010	?UTI	2–18 years	0.94	1	17	0.97	1	72

^a^Calculated values are expressed to 1 significant figure when *n* < 10, to 2 sf when *n* = 10 < 200, and to 3 sf when *n* ≥ 200

^b^Sensitivity evidence levels detailed in text. Summary: 1 = 2 cultures of ≥ 105/ml, 2 = 1 culture or threshold of 104/ml, 3 = included mixed growths or non-uropathogens as culture positive. ^c^Specificity evidence levels = at least one sterile sample

Level-1 sensitivity evidence was provided for 2/11 studies of infants and 5/9 for older children. Six level-2 studies of sensitivity had used a single sample [16, 20, 21, 25, 26, 30], one had used a 10^4^ cfu/ml threshold [22], and two had done both [23, 24]. One grade-3 paper had counted mixed growths [18], and one included *Staphylococcus albus* cultures [17] as positive. In three papers which had previously used lower diagnostic thresholds [16, 20, 21], I recalculated their data using ≥ 10^5^ cfu/ml. I rejected one study that had counted proteus contamination as UTI [31], another because of unacceptable design features [32], one because of selection bias [18], and one because the data was insufficient to reanalyse [33].

### Sensitivity

**Fig. 1:**
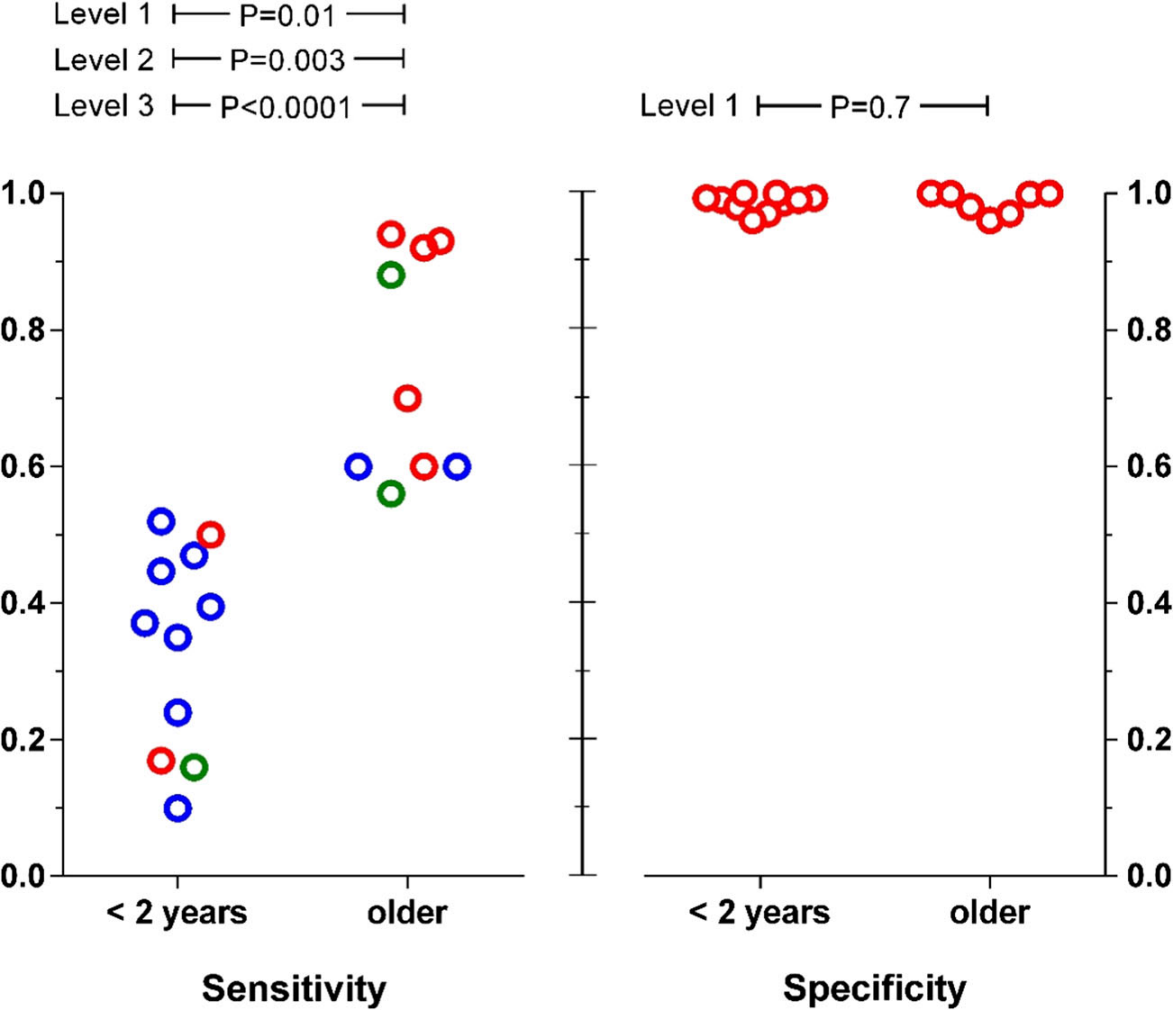
Plot of sensitivity and specificity data for individual studies of using nitrite sticks to detect UTIs in infants aged < 2 years compared with older children. Data quality levels: 1 = red, 2 = blue, 3 = green. The *p* values refer to unpaired *t* test results

**Fig. 2:**
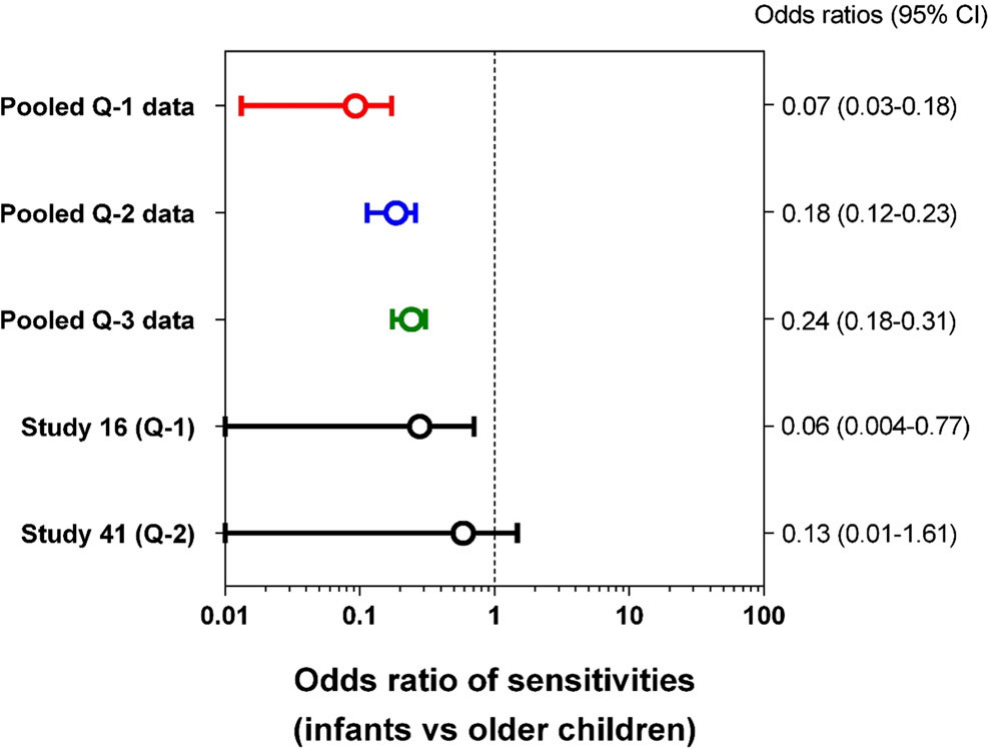
Forest plot of the sensitivity odds ratios and 95% confidence intervals of the ability of nitrite sticks to detect UTIs in infants aged < 2 years compared with older children. Data quality levels for pooled data: 1 = red, 2 = blue, 3 = green. Also, the results are shown for two studies that each tested children in both age groups

The individual study mean sensitivity values were all lower in infants than in older children (see Table 1 and Fig. 1; t, *p* < 0.0001). The same was true for sensitivity data that was pooled from all the studies (Table 2). At level-1 quality, only 7/30 infants with UTIs had positive nitrite sticks, compared to 105/129 older children, giving sensitivity values of 0.23 vs 0.81 (F, *p* < 0.0001). The level-2 and level-3 quality data showed the same relationship (F, *p* < 0.0001 in each case), as shown by a forest plot of their odds ratios (Fig. 2). The two studies which tested both age groups [14, 16] are also shown in this plot.

**Table 2:** Sensitivity and specificity values of the pooled data from studies of nitrite stick testing, according to age range and the evidence quality

Evidence level	Studies of infants				Studies of older children				Difference by age
	Studies		Cases	Sensitivity or specificity	Studies		Cases	Sensitivity or specificity	Contingency table analysis
	(*n*)	(References)	(*n*)		(*n*)	(References)	(*n*)		(*p* value)
Sensitivity									
Level 1 only	2	[14, 19]	30	0.23	5	[12, 14, 27–29]	129	0.81	< 0.0001^F^
Levels 1 and 2	10	[14, 16, 19–26]	1284	0.38	7	[12, 14, 16, 27–30]	153	0.78	< 0.0001^F^
All 3 levels	11	[14, 16, 18–26]	1321	0.38	9	[12, 14, 16, 17, 27–30]	259	0.72	< 0.0001^F^
Specificity									
Level 1	10	[14, 16, 19, 22–26, 31]	1783	0.990	7	[14, 16, 17, 27–30]	5952	0.996	0.002^χ^

^F^Fisher’s exact test. ^*χ*^Chi-square test

### Specificity

Every study had high specificity values (Table 1 and Fig. 1), with no detectable age effect using unpaired *t* testing (t, *p* = 0.95). The pooled specificity values were 0.990 for 1783 infants and 0.996 for 5952 older children (Table 2), equivalent to false-positive rates of 1.0% in infants and 0.4% in the older children (*χ*^2^, *p* < 0.002). This statistically significant difference is too small to be of clinical importance.

### Leaf plot

**Fig. 3:**
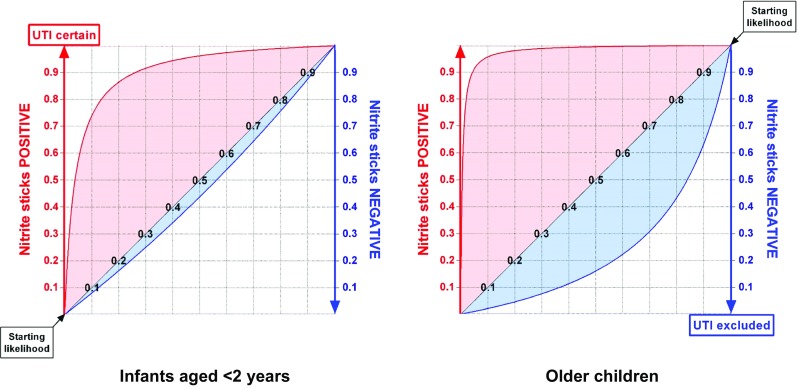
Leaf plots showing the diagnostic value of nitrite stick testing to detect urinary tract infections (UTIs) in infants aged < 2 years and for older children. The vertical increase in height of the red line above the diagonal (vein of the ‘leaf’) shows the impact that a positive culture result has on the probability of that child having a UTI. The vertical drop from the ‘vein’ to the blue line indicates the effect of a negative urine culture on excluding a UTI

Leaf plots aid the interpretation of positive or negative nitrite stick test results, according to the clinical pre-test probability of that individual child having a UTI, which should be read from the appropriate position along the diagonal ‘vein’ of the leaf (Fig. 3) [15]. If nitrite testing was used to screen healthy children for asymptomatic UTI, the pre-test probability would be read from the bottom left of the vein; for a child with an unexplained fever, it would be read about half-way along; and for a febrile child with frequent previous UTIs and offensive-smelling urine, it would be read from near the top right corner. The extra impact of a positive nitrite test is to elevate the chances of a UTI diagnosis to the level of the red line, and of a negative one is to reduce it to the level of the blue line.

The plots in Fig. 3 confirm that a positive nitrite stick test in a child over 2 years old is a powerful indicator that they have a UTI, at almost every level of prior probability, and a fairly powerful indicator in infants. The older children’s plot shows that a negative nitrite result is moderately helpful in ruling out a UTI after 2 years of age, which may be clinically useful as they typically have a low risk of developing kidney scars from a missed first infection [34]. However, the infant plot shows that a negative nitrite stick result has virtually no value for ruling out UTIs in the very young (note the small blue area below the leaf ‘vein’).

## Discussion

### Excluding the diagnosis of a UTI in infants

This reanalysis shows that urine nitrite stick tests miss UTIs in about three quarters of children aged < 2 years, probably due to a combination of low dietary nitrate [10, 11], the instability of their urinary nitrite [11, 13], and urinary frequency [5–7, 33]. This means that it is unsafe to rely on negative nitrite stick test results to decide which infants to treat promptly, which is unfortunate because they are very convenient [5], especially in primary care [35]. Yet, it is important to commence antibiotics before the culture results are available (typically 3 or 4 days later [35]) as this reduces the risk of infants developing kidney scars [3, 4]. Reliable alternative tests that can be carried out at the point-of-care include phase-contrast microscopy of fresh urine [14], or even Gram-staining [22], but another option is to commence antibiotics on clinical suspicion and then discontinue them in culture-negative cases [3]. Older children at particular risk of scarring, for example because they are known to have persistent vesicoureteric reflux [3, 4], should be treated in the same way as infants.

### Study limitations

Calculated sensitivity data for any test will be falsely low if the new method is tested against ‘gold-standard’ techniques which themselves produce false-positive results. This has been the case in some previous nitrite stick studies, where the reference culture data has been less rigorous than the quality level-1 definitions I have used [14]. However, in this meta-analysis, nitrite stick sensitivity was confirmed to be low in infants whichever quality level of evidence we used. The analysis would have been more robust if more than just two source papers had each investigated both infants and older children [14, 16], rather than us having to make age-group comparisons between different publications. However, the difference between the age groups was so large that it was clear despite these shortcomings.

### Age ranges tested

I imposed an arbitrary age cut-off at 2 years, though it is physiologically implausible that the sensitivity of nitrite stick testing would change suddenly at this point. However, it was necessary to define a clinically relevant threshold, and children younger than this are particularly vulnerable to develop scars after a UTI [1, 3, 4]. Other age ranges may also be interesting to investigate. Our previous study [14] suggests that nitrite sticks may be most sensitive in 2 to 9-year-olds (16/17; 0.94), but less sensitive in both infants (3/6; 0.5: F, *p* = 0.04), and in older girls (8/13; 0.62: F, *p* = 0.06) who may acquire UTIs with non-nitrate-reducing *Enterococcus* sp. [36]. It might be easier to investigate how nitrite stick sensitivity varies with age in vitro by inoculating *E. coli* into urine specimens collected from healthy children.

## Conclusions

It is not safe to use nitrite sticks to screen children aged < 2 years for UTIs because they would miss about three quarters of positive cases, though they are useful at some ages, such as between 2 and 9 year of age. This is unfortunate because infants carry the greatest risk of developing kidney scars if they are not treated for UTIs within 3 days. By contrast, a positive nitrite result is very likely to indicate a true UTI at any age.
